# Primary Tumor Characteristics Are Important Prognostic Factors for Sorafenib-Treated Patients with Metastatic Renal Cell Carcinoma: A Retrospective Multicenter Study

**DOI:** 10.1155/2017/9215930

**Published:** 2017-02-07

**Authors:** Sung Han Kim, Sohee Kim, Byung-Ho Nam, Sang Eun Lee, Choung-Soo Kim, Ill Young Seo, Tae Nam Kim, Sung-Hoo Hong, Tae Gyun Kwon, Seong Il Seo, Kwan Joong Joo, Kanghyon Song, Cheol Kwak, Jinsoo Chung

**Affiliations:** ^1^Departments of Urology, Center for Prostate Cancer, National Cancer Center, Goyang, Republic of Korea; ^2^Division of Cancer Epidemiology and Prevention, Research Institute and Hospital, National Cancer Center, Biometric Research Branch, Goyang, Republic of Korea; ^3^Department of Cancer Control and Policy, Graduate School of Cancer Science and Policy, National Cancer Center, Goyang, Republic of Korea; ^4^Department of Urology, Seoul National University Bundang Hospital, Seongnam, Republic of Korea; ^5^Department of Urology, Asan Medical Center, University of Ulsan College of Medicine, Seoul, Republic of Korea; ^6^Department of Urology, Institute of Wonkwang Medical Science, Wonkwang University School of Medicine, Iksan, Republic of Korea; ^7^Department of Urology, Pusan National University Hospital, Busan, Republic of Korea; ^8^Department of Urology, Seoul St. Mary's Hospital, The Catholic University, Seoul, Republic of Korea; ^9^Department of Urology, School of Medicine, Kyungpook National University, Daegu, Republic of Korea; ^10^Department of Urology, Samsung Medical Center, Sungkyunkwan University School of Medicine, Seoul, Republic of Korea; ^11^Department of Urology, Kangbuk Samsung Hospital, Sungkyunkwan University School of Medicine, Seoul, Republic of Korea; ^12^Department of Urology, Korea Cancer Center Hospital, Seoul, Republic of Korea; ^13^Department of Urology, Seoul National University Hospital, Seoul, Republic of Korea

## Abstract

We aimed to identify prognostic factors associated with progression-free survival (PFS) and overall survival (OS) in metastatic renal cell carcinoma (mRCC) patients treated with sorafenib. We investigated 177 patients, including 116 who received sorafenib as first-line therapy, using the Cox regression model. During a median follow-up period of 19.2 months, the PFS and OS were 6.4 and 32.6 months among all patients and 7.4 months and undetermined for first-line sorafenib-treated patients, respectively. Clinical T3-4 stage (hazard ratio [HR] 2.56) and a primary tumor size >7 cm (HR 0.34) were significant prognostic factors for PFS among all patients, as were tumor size >7 cm (HR 0.12), collecting system invasion (HR 5.67), and tumor necrosis (HR 4.11) for OS (*p* < 0.05). In first-line sorafenib-treated patients, ≥4 metastatic lesions (HR 28.57), clinical T3-4 stage (HR 4.34), collecting system invasion (univariate analysis HR 2.11; multivariate analysis HR 0.07), lymphovascular invasion (HR 13.35), and tumor necrosis (HR 6.69) were significant prognosticators of PFS, as were bone metastasis (HR 5.49) and clinical T3-4 stages (HR 4.1) for OS (*p* < 0.05). Our study thus identified a number of primary tumor-related characteristics as important prognostic factors in sorafenib-treated mRCC patients.

## 1. Introduction

Up to one-third of patients with renal cell carcinoma (RCC) present with advanced disease globally, and 20–40% of those who undergo nephrectomy for localized RCC subsequently develop metastases [1]. In the previous era of immunotherapy, the prognosis of patients with unresectable and/or metastatic RCC (mRCC) was dismal, and the average survival was approximately 12 months; only a fraction of patients (10–20%) benefit from cytokine treatment because of limited therapeutic options and the resistance of RCC to conventional chemotherapy [2, 3].

Since 2005, a number of novel targeted therapy (TT) agents that show better efficacy for the treatment of advanced RCC, compared to previous immunochemotherapy agents, have been introduced, and the prognoses of advanced diseases such as mRCC have greatly improved. However, prognoses vary widely, causing clinicians to question the predictive prognostic models of TTs in mRCCs. Various studies have attempted to stratify patients into poor, intermediate, and favorable prognosis groups by investigating multiple risk factors. The Memorial Sloan Kettering Cancer Center (MSKCC) [4] and Heng [5] risk criteria form the bases of prognostic classifications. Several predictive prognostic factors for TTs include laboratory findings, performance and immune status, physical condition, and tumor burden [6–9].

Among the various TTs, sorafenib was one of the first available and globally used tyrosine kinase inhibitors for mRCC patients, with good tolerability and safety. The 2015 guidelines of the National Comprehensive Cancer Network (NCCN) recommended sorafenib as a first-line treatment for patients with relapsed or medically unresectable, predominantly clear cell, stage IV RCC (category 2A) [10]. Many large-scale multicenter studies are currently ongoing in different countries to assess sorafenib's efficacy and safety profile in mRCC patients of different ethnicities using progression-free survival (PFS) and overall survival (OS) as endpoints; these studies seek to identify important prognostic factors in their populations [11–13]. However, few studies have investigated Asian patients with mRCC. Therefore, a representative group of mRCC patients treated with sorafenib was selected from the databases of 11 academic institutions to identify the relevant prognostic factors, including primary tumor-related factors, for PFS and OS. In addition, sorafenib-treated patient survival curves were plotted to compare the prognoses of different risk groups according to the MSKCC [4] and Heng [5] risk criteria.

## 2. Patients and Methods

### 2.1. Patient Selection

A retrospective analysis of 184 clinically diagnosed mRCC patients from 11 Korean academic institutions was performed and included those who had been treated with sorafenib with or without prior systemic therapies between 2006 and 2012. After excluding patients aged <18 years and those with unavailable medical follow-up records, 177 patients were ultimately enrolled after pathological confirmation of RCC in their primary or metastatic site(s) by nephrectomy, metastasectomy, or tumor biopsy. Tumors were stage IV according to the 2009 American Joint Committee on Cancer staging classification.

### 2.2. Treatment and Diagnostic Modalities

Sorafenib treatment was commenced at 400 mg orally twice daily on a continuous basis until disease progression was noted in accordance with the Response Evaluation Criteria in Solid Tumors (RECIST) version 1.0 or the development of intolerance was noted. Tumor response was measured starting at 4–12 weeks after initiation of treatment using RECIST. The primary tumor size was calculated using the contrast phase of baseline computed tomography (CT) images, as was the longest horizontal diameter of the primary unresectable mRCC-affected kidney in situ. For resectable mRCCs, measurements were performed on the pathologic specimen after nephrectomy but before formalin fixation.

Pre- and posttreatment evaluation consisted of a complete history and physical examination, complete blood count, liver and renal function tests, CT of the chest, CT or magnetic resonance imaging of the abdomen and pelvis, and total body bone scintigraphy. Fluorodeoxyglucose positron emission tomography-CT scanning was optional and was performed at each clinician's discretion to evaluate small suspicious multiple metastatic lesions that were not identifiable on CT images. During follow-up, all patients were required to undergo examination by their attending urologists, with work-ups performed according to their respective institutional protocols. Follow-up continued after the termination of treatment until death.

### 2.3. Statistical Methods

PFS was determined from the date of the initiation of sorafenib treatment until documentation of radiologically confirmed disease progression or death from any cause. OS was calculated from the date of initiating sorafenib treatment until death from any cause. All baseline clinicopathological parameters were analyzed as discrete variables with the Chi-square and Wilcoxon rank sum tests as appropriate. The Kaplan-Meier method was used to estimate time-to-event distributions of PFS and OS according to the MSKCC (2002 version) [4] and Heng [5] risk criteria. Univariate and multivariate Cox regression models were employed to identify potential baseline prognostic variables for PFS and OS in all patients as well as in the first-line sorafenib-treated patients separately. Cox regression analysis with Firth's penalized likelihood was used for rare events. Clinically important variables, such as primary tumor-related factors, were subjected to multivariate analysis even if not found to be significant on univariate analyses. All statistical analyses were performed using the STATA statistical software (release 13.1, STATA Inc., TX, USA).

### 2.4. Ethics Statement

This retrospective study was approved by the Institutional Review Board (IRB) of the National Cancer Center (IRB number NCCNCS-11-439) and other participating hospitals. The informed consent requirement was waived by the IRB.

## 3. Results

During a median follow-up of 19.2 months (range, 0.2–62.3 months), the median PFS and OS were 6.4 (range, 5.2–8.9) and 32.6 (range, 27.3–63.8) months for all 177 patients, respectively; for the 116 first-line sorafenib-treated patients, the median PFS and OS were 7.4 (range, 5.5–10.0) months and unattained at 57.8 months, respectively (Table 1). With respect to the best response to sorafenib, the objective response and disease control rates among all 177 patients were 22% and 53.1%, respectively, while the rates among the 116 first-line sorafenib-treated patients were 23.2% and 56%, respectively. All the other baseline demographics, including clinicopathological data and imaging parameters, are described in Table 1.

Significant prognostic factors for PFS and OS were found on multivariate analysis. Clinical T3-4 stage (hazard ratio [HR] 2.56, 95% confidence interval [CI] 1.08–6.09) was a negative predictor of PFS (Table 2), while collecting system invasion (HR 5.67, 95% CI 1.59–22.56) and tumor necrosis (HR 4.11, 95% CI 1.06–21.78) were negative predictors of OS (Table 3) (*p* < 0.05). Conversely, a primary tumor size >7 cm was indicative of significantly better PFS (HR 0.34, 95% CI 0.12–0.98) and OS (HR 0.12, 95% CI 0.02–0.7) (*p* < 0.05; Tables 2 and 3, resp.).

Further multivariate subanalysis of the 116 first-line sorafenib-treated patients revealed that ≥4 lesions at the metastatic sites (HR 28.57, 95% CI 1.74–468.69), clinical T3-4 stage (HR 4.34, 95% CI 1.20–15.71), lymphovascular invasion (HR 13.35, 95% CI 1.91–93.37), and necrosis within the primary kidney tumor (HR 6.69, 95% CI 2.06–21.73) were significantly poor prognostic indicators of PFS, whereas collecting system invasion (HR 0.07, 95% CI 0.01–0.55) was the sole favorable parameter (*p* < 0.05; Table 4). Furthermore, bone metastasis (HR 5.49, 95% CI 1.62–18.65) and clinical T3-4 stage (HR 4.1, 95% CI 1.08–15.51) were significantly negative prognostic parameters of OS (*p* < 0.05; Table 5).

**Figure 1:**
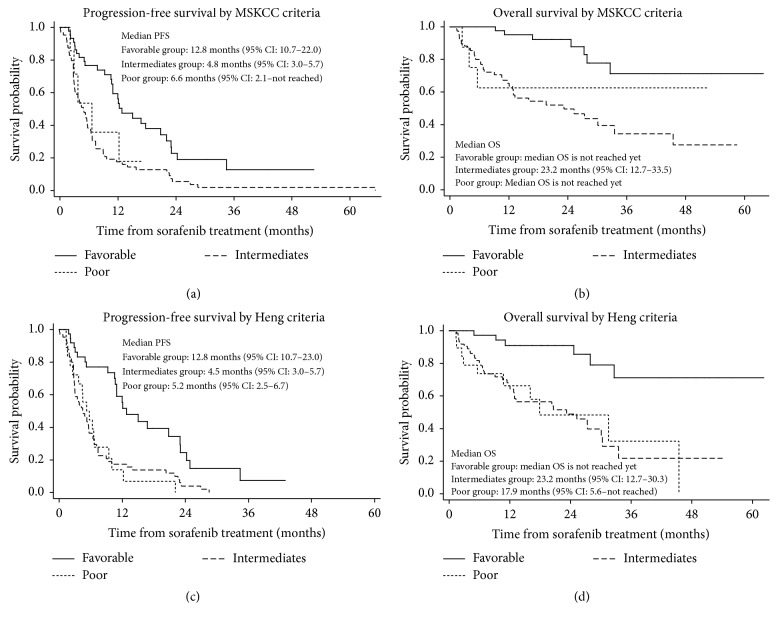
Kaplan-Meier analysis of progression-free survival (a, c) and overall survival (b, d) with log-rank tests for all sorafenib-treated patients (*N* = 177) according to the Memorial Sloan Kettering Cancer Center (MSKCC) (a, b) and Heng (c, d) risk groups. PFS, progression-free survival; OS, overall survival; CI, confidence interval.

**Figure 2:**
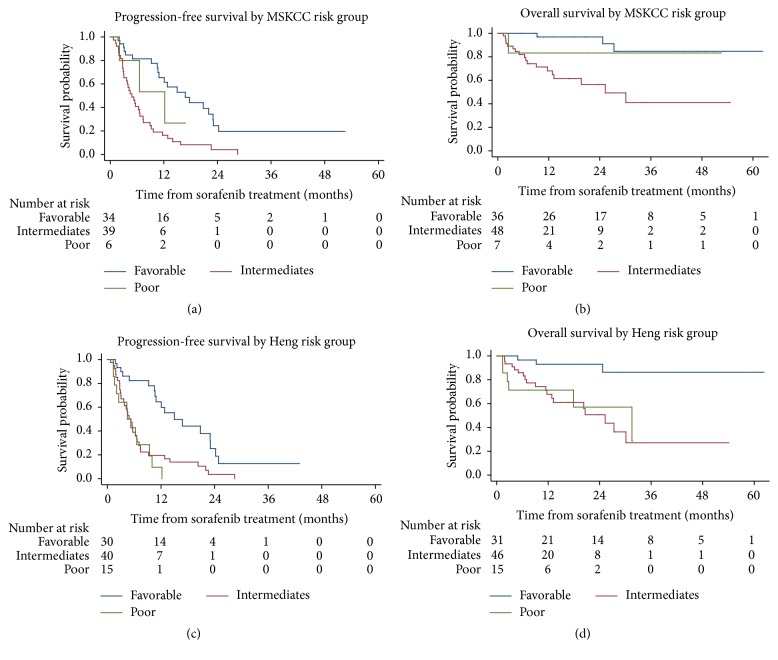
Kaplan-Meier analysis of progression-free survival and overall survival with log-rank tests for first-line sorafenib-treated patients (*N* = 116) according to the Memorial Sloan Kettering Cancer Center (MSKCC) (a) and Heng (b) risk groups. PFS, progression-free survival; OS, overall survival; CI, confidence interval.

**Figure 3:**
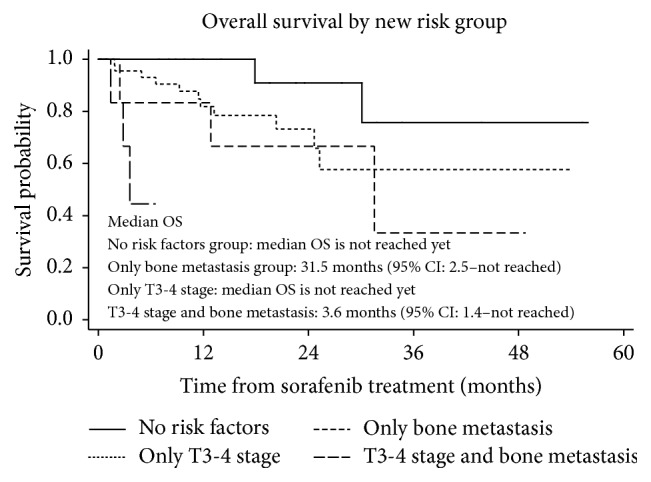
Comparison of overall survival (OS) according to the presence of bone metastasis and/or T3-4 stage, which were poor prognostic factors of OS in first-line sorafenib-treated patients.

Kaplan-Meier analyses revealed that patients categorized according to MSKCC versus Heng criteria had different PFS and OS. The MSKCC intermediate group (i.e., those with 1-2 risk factors) had the worst PFS (4.8 months; versus 6.6 months for the poor group and 12.8 months for the favorable group; *p* < 0.001) and OS (23.2 months; versus both the poor and favorable groups where OS was yet undetermined, *p* < 0.002) when considering all 177 sorafenib-treated patients (Figure 1). When categorized according to the Heng risk criteria, the intermediate group also had worse PFS (4.5 months) than the favorable and poor groups (12.8 and 5.2 months, resp.); however, the OS in the intermediate group (23.2 months) was similar to that of the poor group (17.9 months) when compared to the reference group (i.e., the favorable group; OS yet undetermined) (*p* < 0.001) (Figure 1). Additional subanalyses of the first-line sorafenib-treated patients with the MSKCC and Heng risk criteria were performed using the Kaplan-Meier and log-rank tests, revealing that similar patterns of OS and PFS were observed according to the MSKCC criteria, but not according to the Heng criteria (Figure 2). Furthermore, we compared the OS of the first-line sorafenib groups according to the presence of bone metastasis and/or T3-4 stage, which we had found to be poor prognostic factors; significant differences in OS were observed (*p* = 0.0003; Figure 3).

## 4. Discussion

Globally, sorafenib has proven to be tolerable, safe, and effective for treating mRCC patients. It is essential that the prognostic factors of PFS and OS be clarified in order to guide patient care and yield the best therapeutic response. Several previous studies suggested various parameters and models for classifying mRCC patients into favorable, intermediate, and poor risk groups according to the number of survival risk factors [2, 5, 8, 9, 14, 15]. Using a nationwide Korean kidney cancer database encompassing 11 Korean academic institutions, this study clarified the prognostic importance of several independent primary tumor characteristics, as well as of the extent of disease, in sorafenib-treated mRCC patients with or without prior systemic therapies. Specifically, clinical T3-4 stage was a negative prognosticator of PFS, as were collecting system invasion and tumor necrosis for OS. Conversely, a primary tumor size >7 cm was a favorable prognostic factor for both PFS and OS.

Previous studies suggested that the intermediate risk group, as defined by the MSKCC and Heng criteria, had multiple pitfalls because of the uneven distribution of a large number of diverse patients; this produced heterogeneous outcomes that necessitated further stratification [16, 17]. We encountered similar findings as well, with the intermediate group having disproportionately worse PFS (4.8 months) and OS (23.2 months) than the poor risk groups; this was also the case when using the Heng risk model. In addition, significantly different survival outcomes were observed for groups stratified according to the MSKCC and Heng risk criteria when analyzing all patients as well as only first-line sorafenib-treated patients (*p* < 0.05, Figures 1 and 2). The Heng criteria appeared to be slightly more correlative with OS than the MSKCC criteria, which is consistent with a previous Korean study with sunitinib that showed the Heng risk model to have slightly better discriminatory ability than the MSKCC model [17].

Among the significant prognostic factors for PFS and OS in all patients, primary tumor size had an HR <1.0 on both univariate and multivariate analyses, indicating that mRCC patients with greater sized primary tumors, especially those >7 cm, responded better to sorafenib and had more favorable prognoses in our study. This implies that smaller sized renal tumors with metastatic lesions might be more aggressive than larger tumors. Subgroup analysis that stratified tumor size into four groups (<4 cm, 4–7 cm, 7–10 cm, and >10 cm) was performed on 140 patients with previous nephrectomies; these 4 subgroups were statistically correlated with low (1-2) and high (3-4) Fuhrman nuclear grades. A greater proportion of small tumors were of higher tumor grades, albeit without statistical significance owing to a small sample size (*p* = 0.068; odds ratio 0.675; data not shown).

In addition to a primary tumor size >7 cm, other significant factors determining survival among the 177 patients were clinical T3-4 stage (for PFS and OS), collecting system invasion (for OS), and tumor necrosis (for OS) within the primary renal tumor. These data corresponded to previous studies of invasive and advanced RCC, where clinical T3-4 stage, aggressive characteristics such as collecting system invasion [18, 19], and faster growth rates with tumor necrosis [20–22] reflected advanced disease states with a negative impact on survival. However, none of these previous reports included TT agent-treated mRCC patients; this is the first study to show that certain primary tumor-related factors were significant indicators of prognosis in TT agent-treated mRCC patients, particularly those receiving sorafenib.

Separate analysis of prognostic factors for PFS and OS in 116 patients with first-line sorafenib treatment showed that clinical T3-4 stages (HR 4.34), collecting system invasion (HR 2.11 on univariate analysis and HR 0.07 on multivariate analysis), tumor necrosis (HR 6.69), lymphovascular invasion (HR 13.35), and metastatic sites ≥4 lesions (HR 28.57) were significant predictors of PFS; this is also the first study to show that collecting system invasion, tumor necrosis, and lymphovascular invasion are significant prognostic factors in naive sorafenib-treated mRCC patients. However, the HRs for collecting system invasion were not correlated on univariate versus multivariate analyses because of multicollinearity between collecting system invasion, lymphovascular invasion, and tumor necrosis. When excluding both lymphovascular invasion and tumor necrosis, collecting system invasion was found to be a poor prognostic factor for PFS in the first-line sorafenib group (HR: 2.24; data not shown).

Bone metastasis and clinical T3-4 stages were the only significant predictors of OS in first-line sorafenib-treated patients. The OS curves were compared among patients with poor prognostic factors (i.e., those with bone metastasis, T3-4 stage, or both); there were significant differences in OS rates among each of these groups. The presence of bone metastases in mRCC patients has been shown to be a poor prognostic factor despite sorafenib treatment [23]; therefore, mRCC patients with newly diagnosed bone metastases should be considered for active treatment of the metastatic bone lesion with either surgery or radiation therapy in combination with systemic medical therapy. This can improve both the OS and quality of life for mRCC patients treated with sorafenib.

First-line sorafenib-treated patients were separately subjected to subanalyses to identify additional significant prognostic factors of OS, since multianalyses with numerous interrelated parameters, or those conducted with missing values and multicollinearities from retrospectively collected data, may have decreased statistical power and significance. Multivariate Cox statistics with backward selection were performed in 2 independent analyses while dividing the variables into (1) laboratory parameters and risk criteria and (2) primary pathological tumor-related factors (including TNM staging). Our results were consistent with those of previous studies [23–26]; we found that bone metastasis (HR 4.6) and clinical T3-4 stage (HR 3.8) were significant negative prognostic factors for survival and that female sex (HR 0.16), thrombocytosis (HR 0.23), lymphovascular invasion (HR 0.20), and primary tumor size >7 cm (HR 0.15) were significant positive prognostic factors (*p* < 0.05, data not shown).

The prognostic factors identified in this study collectively indicated that the therapeutic response to sorafenib could be more dependent on the characteristics of the primary renal tumor and the overall extent of the disease than on general health status. A greater primary tumor size with smaller necrotic areas, the absence of lymphovascular and collecting system invasion within the primary tumor, and less metastatic lesions without bone involvement are expected to reflect better prognosis with sorafenib treatment, whether before or after nephrectomy or metastasectomy [15, 27, 28]. Additionally, Araki et al. emphasized the importance of primary tumor characteristics, especially tumor growth patterns, and also found that the Fuhrman nuclear grade, presence of a sarcomatoid component, lymphovascular invasion, tumor necrosis, growth pattern, and other pathological parameters of the primary tumor were potentially useful prognostic indicators, as they could be assessed easily at the time of nephrectomy [22]. The importance of primary tumor characteristics and disease extent in this study was noteworthy because sorafenib is considered systemically less potent than other similarly acting tyrosine kinase inhibitors, although with more tolerability, fewer dose reductions, and/or less severe adverse events [12]. Furthermore, the prognostic significance of histology (clear and non-clear cell pathology) was generally not found to be of statistical significance; the small number of non-clear cell RCC patients in this study may not have been sufficient to influence the statistical analyses, and sorafenib has multiple inhibitory mechanisms involving multiple pathways [13].

This study harbored potential selection bias because of its retrospective nature as well as incomplete data collection, no further treatment information on other second-line and/or salvage therapy following treatment failure with sorafenib, and heterogeneity of clinicians' use of sorafenib due to this being a multicenter study. Further large-scale, prospectively designed studies are necessary to confirm our findings. These would include comparing the therapeutic effectiveness of sorafenib to other targeted therapies in patients with primary mRCC tumors sized >7 cm, as patients whose primary tumors were T2 stage were good candidates for sorafenib therapy in contrast to patients with T3-4 stage mRCCs. Moreover, this study showed that primary tumor size and T3-4 stage were significant prognostic factors in terms of PFS and OS, not only for sorafenib-treated patients but also for mRCC patients in general.

Despite these limitations, the current study is the first large-scale multicenter study to reveal the prognostic factors of primary tumor-related characteristics associated with first-line sorafenib treatment in terms of PFS and OS in Korean mRCC patients. Our results might be of relevance to poor risk patients without aggressive primary tumor characteristics (such as clinical T3-4 stage and bone metastasis) who may be candidates for sorafenib therapy, especially as the NCCN guideline does not currently specify the patients for whom sorafenib is indicated [10]. Older patients and those with high underlying comorbidities, hepatic dysfunction, and major toxic adverse effects with sunitinib and pazopanib might be indicated for sorafenib therapy [11, 12, 29, 30]. Although the use of sorafenib as a first-line treatment is limited, and resulting PFS and OS rates are inferior to those observed with other TTs such as sunitinib, pazopanib, and combination of bevacizumab and interferon alpha, proper selection of patients as recommend by category 2A of the NCCN guideline may result in more positive prognoses following sorafenib therapy [4, 5, 10–12].

## 5. Conclusions

In this study of the long-term efficacy of sorafenib as an overall or first-line therapy, we showed that primary renal tumor-related characteristics, as well as the extent of disease, were significant prognostic indicators in sorafenib-treated Korean patients with mRCC.

## Figures and Tables

**Table 1:** Baseline demographics.

Parameter	Overall (*N* = 177)	First-line (*N* = 116)
	*N* (percentage) or median (range)	*N* (percentage) or median (range)
Gender (male/female)	136/41 (76.8/23.2)	91/25 (78.4/21.6)
Age (years)	62.0 ± 10.9	63.8 ± 10.5
Follow-up duration (months)	19.2 (0.2–63.8)	18 (0.2–57.8)
Treatment duration (weeks)	20.1 (1–216)	23.9 (4.4–176)
Body mass index (kg/cm^2^)	23.3 (14.5–37.2)	23.1 (14.5–37.2)
Comorbidity		
Diabetes	42 (23.7)	29 (25.0)
Hypertension	75 (42.4)	49 (42.2)
Cerebrovascular accident (CVA)	5 (2.8)	2 (1.7)
Cardiovascular disease	6 (3.4)	6 (5.2)
Liver disease	3 (1.7)	2 (1.7)
Renal disease	8 (4.5)	5 (4.3)
Deep vein thrombosis (DVT)	1 (0.6)	1 (0.9)
Hypercholesterolemia	3 (1.7)	3 (2.6)
Presenting symptom	101 (57.1)	62 (53.4)
Incidental renal mass	11 (6.2)	9 (7.8)
Symptom developed	83 (46.9)	52 (44.8)
Other	7 (4.0)	1 (0.9)
Body surface area (m^2^)		
≤1.7	74 (41.8)	52 (44.8)
>1.7	87 (49.2)	60 (51.7)
Unknown	16 (9.0)	4 (3.4)
ECOG		
0	110 (62.1)	59 (50.9)
1	54 (30.5)	46 (39.7)
2	9 (5.1)	8 (6.9)
3	1 (0.6)	1 (0.9)
Unknown	3 (1.7)	2 (1.7)
Karnofsky performance score		
>80	107 (60.5)	63 (54.3)
50–80	22 (12.4)	22 (19.0)
<50	7 (4.0)	6 (5.2)
Unknown	41 (23.2)	25 (21.6)
MSKCC risk criteria		
Favorable	49 (27.7)	33 (28.4)
Intermediate	82 (46.3)	48 (41.4)
Poor	9 (5.1)	7 (6.0)
Unknown	37 (20.9)	28 (24.1)
Heng risk criteria		
Favorable	39 (22.0)	29 (25.0)
Intermediate	78 (44.1)	46 (39.7)
Poor	19 (10.7)	15 (12.9)
Unknown	41 (23.2)	26 (22.4)
Prior surgical therapy		
Nephrectomy (radical/partial/embolization)	150 (135/5/10) [84.7 (76.3/2.8/5.6)]	99 (87/5/7) [85.3 (75/4.3/6.0)]
Metastasectomy	44 (24.9)	26 (22.4)
Prior systemic therapy	56 (31.6)	0
Immuno/chemo/sunitinib therapy	33 (18.6)/4 (2.3)/19 (10.7)	—
Number of metastatic sites (18)		
1 organ	94 (53.1)	65 (56.0)
2 organs	44 (24.9)	27 (23.3)
3 organs	19 (10.7)	10 (8.6)
≥4 organs	8 (4.5)	4 (3.4)
Unknown	12 (6.8)	10 (8.6)
Metastatic sites	3 (1–5)	3 (1–5)
Brain	42 (23.7)	33 (28.4)
Bone	38 (21.5)	23 (19.8)
Liver	17 (9.6)	9 (7.8)
Lung	124 (70.1)	81 (69.8)
Lymph node	34 (19.2)	23 (19.8)
Pancreas	8 (4.5)	7 (6.0)
Kidney, contralateral	7 (4.0)	5 (4.3)
Other	30 (16.9)	17 (14.7)
Primary kidney tumor-related parameter		
Size of primary tumor (cm)	8 (1–117)	8 (1–117)
Collecting system invasion	28 (15.8)	16 (13.8)
Capsule invasion	36 (20.3)	24 (20.7)
Lymphovascular invasion	34 (19.2)	29 (25.0)
Tumor necrosis	46 (26.0)	31 (26.7)
TNM stage		
T1	25 (14.1)	16 (13.8)
T2	35 (19.8)	25 (21.6)
T3	74 (41.8)	54 (46.6)
T4	8 (4.5)	8 (6.9)
T*x*	35 (19.8)	13 (11.2)
N1	27 (15.3)	22 (19.0)
M1	131 (74.0)	85 (73.3)
Fuhrman grade		
1	5 (2.8)	2 (1.7)
2	39 (22.0)	26 (22.4)
3	69 (39.0)	47 (40.5)
4	36 (20.3)	23 (19.8)
Unknown	28 (15.8)	18 (15.5)
Histology		
Clear cell, pure	159 (89.8)	110 (94.8)
Non-clear cell	3 (1.7)	1 (0.9)
Unknown	15 (8.5)	5 (4.3)
Best overall response (CR + PR + SD)	94 (53.1)	65 (56.0)
Complete remission	6 (3.4)	4 (3.4)
Partial response	33 (18.6)	23 (19.8)
Stable disease	55 (31.1)	38 (32.8)
Progressive disease^*∗*^	83 (46.9)	51 (44.0)
Progression-free survival (median months)	6.4 (5.2–8.9)	7.4 (5.5–10.0)
Overall survival (median months)	32.6 (27.3–63.8)	NR
Survival	114 (64.4)	85 (73.3)
Cancer-specific death	51 (28.8)	24 (20.7)

^*∗*^Progressive disease = progressive disease + not evaluated disease.

CR, complete response; ECOG, Eastern Cooperative Oncology Group; MSKCC, Memorial Sloan Kettering Cancer Center; NR, not yet reached; PR: partial response; SD, stable disease.

**Table 2:** Multivariate analyses of prognostic factors for progression-free survival in all sorafenib-treated patients (*N* = 177).

Category	Univariate analysis of PFS			Multivariate analysis of PFS		
	Hazard ratio	*p* value	Confidence interval	Hazard ratio	*p* value	Confidence interval
Gender, female	0.76	0.228	0.48–1.19			
Age ≥65 years	1.10	0.620	0.76–1.59			
KPS <80%	1.15	0.654	0.63–2.09			
LDH >1.5x ULN	1.32	0.511	0.57–3.04			
Hemoglobin <LLN	1.62	0.012	1.11–2.37	0.86	0.724	0.36–2.02
cCa >10 mg/dL	0.88	0.769	0.39–2.02			
Time from diagnosis to treatment <1 year	1.99	<0.001	1.37–2.90	1.32	0.531	0.56–3.12
Leukocytosis	1.82	0.114	0.87–3.80			
Thrombocytosis	3.24	0.002	1.54–6.82	0.70	0.677	0.13–3.75
Hypoalbuminemia	1.23	0.541	0.64–2.36			
Prior nephrectomy	0.83	0.751	0.26–2.63			
Prior metastasectomy	0.93	0.723	0.61–1.41			
Brain metastasis	0.97	0.881	0.64–1.47			
Bone metastasis	1.38	0.117	0.92–2.07			
Liver metastasis	2.01	0.025	1.09–3.71	1.66	0.377	0.54–5.08
Lung metastasis	0.77	0.192	0.52–1.14			
Lymph node metastasis	1.24	0.340	0.80–1.93			
Pancreas	1.17	0.673	0.57–2.40			
Contralateral kidney	0.22	0.036	0.05–0.91	0.29	0.286	0.03–2.80
Metastatic sites						
1	1.00					
2-3	1.04	0.845	0.71–1.52			
≥4	1.29	0.532	0.58–2.85			
T stage						
T1-2	1.00			1.00		
T3-4	1.55	0.028	1.05–1.52	2.56	0.034	1.08–6.09
N1	2.26	0.001	1.38–2.85	1.31	0.714	0.31–5.54
M1	1.98	0.033	1.06–2.30	0.84	0.721	0.31–2.22
Fuhrman grade						
1-2	1.00					
3-4	0.96	0.857	0.63–3.68			
Tumor-related factor						
Primary tumor size						
<4 cm	1.00			1.00		
4–7 cm	0.92	0.811	0.45–1.87	0.54	0.281	0.18–1.66
>7 cm	0.78	0.447	0.40–1.49	0.34	0.045	0.12–0.98
Collecting system invasion	1.67	0.045	1.01–2.74	0.73	0.582	0.24–2.23
Capsule invasion	1.01	0.981	0.62–1.64			
Lymphovascular invasion	2.13	0.004	1.27–3.57	2.01	0.324	0.5–8.09
Tumor necrosis	2.13	0.003	1.30–3.47	2.36	0.055	0.98–5.65
Immunotherapy	1.00					
Other therapy	0.96	0.858	0.62–1.5			

KPS, Karnofsky performance status; LDH, lactate dehydrogenase; ULN, upper limit normal; LLN, lower limit normal; cCa, corrected calcium.

**Table 3:** Multivariate analyses of prognostic factors for overall survival in all sorafenib-treated patients (*N* = 177).

Parameter	Univariate analysis of OS			Multivariate analysis of OS		
	Hazard ratio	*p* value	Confidence interval	Hazard ratio	*p* value	Confidence interval
Gender, female	0.81	0.507	0.44–1.5			
Age ≥65 years	1.05	0.863	0.62–1.77			
KPS <80%	1.14	0.756	0.49–2.67			
LDH >1.5x ULN	1.62	0.311	0.64–4.09			
Hemoglobin <LLN	2.72	<0.001	1.61–4.61	1.54	0.527	0.39–5.77
cCa >10 mg/dL	0.91	0.861	0.33–2.53			
Time from diagnosis to treatment <1 year	1.63	0.072	0.96–2.78			
Leukocytosis	1.03	0.963	0.25–4.30			
Thrombocytosis	7.52	<0.001	3.04–18.63	1.58	0.787	0.01–21.63
Hypoalbuminemia	1.56	0.270	0.71–3.45			
Prior nephrectomy	0.54	0.396	0.13–2.24			
Prior metastasectomy	0.71	0.305	0.37–1.36			
Brain metastasis	1.07	0.813	0.60–1.92			
Bone metastasis	2.16	0.004	1.28–3.66	2.71	0.104	0.81–8.61
Liver metastasis	2.79	0.005	1.36–5.72	2.39	0.318	0.42–14.09
Lung metastasis	1.22	0.505	0.68–2.18			
Lymph node metastasis	1.43	0.225	0.80–2.57			
Pancreas	0.77	0.719	0.19–3.16			
Contralateral kidney	1.02	0.978	0.25–4.19			
Metastatic sites						
1	1.00					
2-3	1.83	0.024	1.08–4.65	1.12	0.885	0.24–4.74
≥4	4.20	0.004	1.59–14.11	1.63	0.663	0.13–12.07
T stage						
T1-2	1.00					
T3-4	1.76	0.068	0.96–3.22			
N1	1.68	0.193	0.77–3.69			
M1	5.70	0.016	1.38–23.45	0.87	0.888	0.15–9.29
Fuhrman grade						
1-2	1.00					
3-4	1.19	0.594	0.63–2.26			
Primary tumor size						
<4 cm	1.00					
4–7 cm	1.07	0.897	0.38–3.04	0.47	0.358	0.09–2.45
> 7cm	0.83	0.705	0.31–1.62	0.12	0.020	0.02–0.7
Collecting system invasion	2.51	0.012	1.22–3.62	5.67	0.008	1.59–22.56
Capsule invasion	0.79	0.555	0.36–3.05			
Lymphovascular invasion	1.37	0.448	0.61–2.22			
Tumor necrosis	4.32	<0.001	1.83–5.16	4.11	0.041	1.06–21.78
Immunotherapy	1.0					
Other therapy	0.71	0.232	0.41–1.72			

KPS, Karnofsky performance status; LDH, lactate dehydrogenase; ULN, upper limit normal; LLN, lower limit normal; cCa, corrected calcium.

**Table 4:** Multivariate analyses of prognostic factors for progression-free survival in first-line sorafenib-treated patients (*N* = 116).

Parameter	Univariate analysis of PFS			Multivariate analysis of PFS		
	Hazard ratio	*p* value	Confidence interval	Hazard ratio	*p* value	Confidence interval
Gender, female	0.56	0.066	0.30–1.04			
Age ≥65 years	1.04	0.859	0.66–1.66			
KPS <80%	1.87	0.062	0.97–3.61			
LDH >1.5x ULN	0.70	0.625	0.17–2.90			
Hemoglobin <LLN	1.78	0.019	1.10–2.87	0.41	0.220	0.10–1.70
cCa >10 mg/dL	0.83	0.684	0.33–2.06			
Time from diagnosis to treatment <1 year	2.88	0.000	1.72–4.81	1.90	0.242	0.65–5.59
Leukocytosis	2.35	0.032	1.08–5.13	2.41	0.274	0.50–11.61
Thrombocytosis	4.06	0.001	1.78–9.30	0.83	0.875	0.09–8.02
Hypoalbuminemia	1.93	0.089	0.91–4.10			
Prior nephrectomy	1.98	0.499	0.27–14.33			
Prior metastasectomy	0.89	0.683	0.52–1.53			
Brain metastasis	1.04	0.867	0.63–1.73			
Bone metastasis	1.65	0.066	0.97–2.81			
Liver metastasis	2.16	0.058	0.98–4.80			
Lung metastasis	0.72	0.187	0.44–1.17			
Lymph node metastasis	1.82	0.040	1.03–3.23	1.98	0.365	0.45–8.72
Pancreas	1.16	0.708	0.53–2.56			
Contralateral kidney	0.23	0.149	0.03–1.69			
Metastatic sites						
1	1.00			1.00		
2-3	1.28	0.320	0.78–2.10	0.80	0.783	0.17–3.80
4	3.96	0.012	1.35–11.56	28.57	0.019	1.74–468.69
T stage						
T1-2	1.00			1.00		
T3-4	1.97	0.008	1.19–3.26	4.34	0.025	1.20–15.71
N1	2.66	0.001	1.51–4.68	0.75	0.837	0.05–11.95
M1	2.08	0.044	1.02–4.22	0.75	0.674	0.20–2.86
Fuhrman grade						
1-2	1.00					
3-4	0.98	0.955	0.57–1.71			
Primary tumor size						
<4 cm	1.00					
4–7 cm	0.87	0.737	0.37–2.00	0.37	0.288	0.06–2.30
>7 cm	0.85	0.690	0.39–1.86	0.31	0.186	0.05–1.76
Collecting system invasion	2.11	0.026	1.09–4.07	0.07	0.012	0.01–0.55
Capsule invasion	1.05	0.869	0.59–1.88			
Lymphovascular invasion	2.41	0.004	1.33–4.37	13.35	0.009	1.91–93.37
Tumor necrosis	2.01	0.022	1.10–3.65	6.69	0.002	2.06–21.73

KPS, Karnofsky performance status; LDH, lactate dehydrogenase; ULN, upper limit normal; LLN, lower limit normal; cCa, corrected calcium.

**Table 5:** Multivariate analyses of prognostic factors for overall survival in first-line sorafenib-treated patients (*N* = 116).

	Univariate analysis of OS			Multivariate analysis of OS		
Category	Hazard ratio	*p* value	95% confidence interval	Hazard ratio	*p* value	95% confidence interval

Gender, female	0.34	0.076	0.10–1.12			
Age ≥65 years	1.13	0.736	0.55–2.36			
KPS <80%	1.55	0.374	0.59–4.07			
LDH >1.5x ULN	0.64	0.659	0.09–4.74			
Hemoglobin <LLN	2.10	0.044	1.02–4.30	1.14	0.824	0.36–3.67
cCa >10 mg/dL	0.53	0.390	0.13–2.24			
Time from diagnosis to treatment <1 year	2.16	0.048	1.01–4.63	1.36	0.584	0.45–4.07
Leukocytosis	1.36	0.685	0.31–5.88			
Thrombocytosis	8.95	0.000	3.08–26.05	4.92	0.068	0.89–27.30
Hypoalbuminemia	2.66	0.035	1.07–6.62	1.94	0.337	0.50–7.46
Prior nephrectomy	0.69	0.714	0.09–5.11			
Prior metastasectomy	0.86	0.748	0.34–2.17			
Brain metastasis	1.18	0.674	0.55–2.50			
Bone metastasis	3.25	0.001	1.57–6.73	5.28	0.015	1.38–20.19
Liver metastasis	2.55	0.087	0.87–7.44			
Lung metastasis	0.83	0.627	0.39–1.76			
Lymph node metastasis	1.71	0.192	0.76–3.86			
Pancreas	0.56	0.563	0.08–4.08			
Contralateral kidney	0.79	0.814	0.11–5.78			
Metastatic sites						
1	1.00					
2-3	1.74	0.150	0.82–3.70			
≥4	2.89	0.160	0.66–12.66			
T stage						
T1-2	1.00			1.00		
T3-4	2.45	0.050	1.00–6.01	4.10	0.038	1.08–15.51
N1	2.14	0.120	0.82–5.56			
M1	7.15	0.054	0.97–52.98			
Fuhrman grade						
1-2	1.00					
3-4	1.85	0.264	0.63–5.45			
Tumor-related factor						
Primary tumor size						
<4 cm	1.00			1.00		
4–7 cm	1.08	0.909	0.29–4.00	0.70	0.633	0.16–3.02
>7 cm	0.81	0.751	0.22–2.95	0.31	0.142	0.07–1.48
Collecting system invasion	2.31	0.130	0.78–6.85			
Capsule invasion	0.69	0.483	0.25–1.92			
Lymphovascular invasion	2.45	0.068	0.94–6.39			
Tumor necrosis	2.75	0.061	0.95–7.93			

KPS, Karnofsky performance status; LDH, lactate dehydrogenase; ULN, upper limit normal; LLN, lower limit normal; cCa, corrected calcium.
